# Efficiency of Plasmon-Induced Dual-Mode Fluorescence Enhancement upon Two-Photon Excitation

**DOI:** 10.3390/nano11123334

**Published:** 2021-12-08

**Authors:** Maria A. Shokova, Vladimir E. Bochenkov

**Affiliations:** Department of Chemistry, Lomonosov Moscow State University, 119991 Moscow, Russia; mariia.shokova@chemistry.msu.ru

**Keywords:** LSPR, plasmon-enhanced fluorescence, silver nanobar, two-photon excitation, FDTD, near-infrared fluorescent proteins

## Abstract

Anisotropic noble metal nanoparticles supporting more than one localized surface plasmon resonance can be tailored for efficient dual-mode fluorescence enhancement by ensuring an adequate coupling to both absorption and emission bands of fluorophores. This approach is naturally extended to two-photon excitation fluorescence, where a molecule is excited by simultaneous nonlinear absorption of two photons. However, the relative impact of plasmon coupling to excitation and emission on the overall fluorescence enhancement can be very different in this case. Here, by using the finite-difference time-domain method, we study the two-photon excitation fluorescence of near-infrared fluorescent protein (NirFP) eqFP670, which is the most red-shifted NirFP to date, in proximity to a silver nanobar. By optimizing the length and aspect ratio of the particle, we reach a fluorescence enhancement factor of $10^{3}$. We show that the single mode coupling regime with highly tuned near-field significantly outperforms the dual-mode coupling enhancement. The plasmon-induced amplification of the fluorophore’s excitation rate becomes of utmost importance due to its quadratic dependence on light intensity, defining the fluorescence enhancement upon two-photon excitation. Our results can be used for the rational design of hybrid nanosystems based on NirFP and plasmonic nanoparticles with greatly improved brightness important for developing whole-body imaging techniques.

## 1. Introduction

In the past few decades, fluorescence microscopy has become one the most widely used techniques in biological imaging due to its ability to selectively reveal otherwise hidden objects of interest inside living cells and organisms [1]. Along with advances in tailoring illuminating beams and emitted light as well as synthesis of a variety of fluorescent probes, the fast progress of fluorescence microscopy has been catalyzed by the discovery of the green fluorescent protein and its variants [2]. These biomolecules, initially found in marine species, are now extensively used as genetically encoded fluorescent tags to highlight virtually any protein of interest [3].

New variants of fluorescence microscopy are being continuously developed to improve spatial resolution, to increase sensitivity and to lower the detection limit of the method. One of the most successful approaches is based on two-photon excitation resulting from simultaneous absorption of two photons with lower energy [4]. It provides a number of improvements, including reduced background absorption and deeper penetration of near-infrared light in biological tissues, good 3D focusing due to a quadratic power dependence of excitation on light intensity, high contrast due to low background absorption and large spectral separation between excitation and emission.

Two-photon excitation fluorescence microscopy is particularly important for deep tissue imaging, since applications of fluorescent proteins are constrained by the opacity of tissues to excitation and emission light below 600 nm. Fluorescent proteins with excitation and emission wavelengths in the red and near-infrared range are therefore of high demand for whole body and multicolor imaging. The eqFP670 protein is currently the most red-shifted fluorescent protein, with excitation and emission peaks at 605 nm and 670 nm, respectively [5]. The protein does not exhibit residual short wavelength fluorescence, in contrast for example to E2-Crimson ($\lambda_{ex}/\lambda_{em}$ = 611/646 nm) [6] and mNeptune ($\lambda_{ex}/\lambda_{em}$ = 600/650 nm) [7]. It is also characterized by high pH stability and extremely high photostability, allowing for accumulation of the fluorescent signal over long exposure times. The eqFP670 protein is particularly promising for developing two-photon excitation fluorescent probes, since it has a broad TPA band with absorption maximum at 1110 nm [8] and near-infrared emission at 670 nm [5]. Both bands fall within the two biological transparency windows (650–950 nm and 1000–1350 nm) [9]. However, the protein has relatively weak brightness, which hinders its broad practical application.

Fluorophore brightness can be significantly improved by its coupling to metal nanostructures, which support a localized surface plasmon resonance (LSPR) [10,11]. Metal nanostructures act as a nanoantenna, boosting the efficiency of excitation transfer to and from the fluorophore. An overlap between the LSPR peak with the absorption and/or emission band of the fluorophore is crucial. Multi-modal plasmonic systems with several LSPR modes, such as nanorods, provide means to independently couple different modes to fluorophore’s excitation and emission by tuning the corresponding plasmon resonances [12,13].

The capability of plasmonic nanoparticles to focus light in tiny volumes with manifold amplification of electric field is especially promising for enhancing efficiency of two-photon absorption (TPA) processes [14]. In addition, tunable multi-modal plasmonic systems offer the possibility to be coupled to both TPA and emission bands of the fluorophore. To date, several geometries of plasmonic nanoparticles suitable for multi-modal fluorescence enhancement with two-photon excitation have been studied, including nanocylinders [15,16], nanorods [17] and nanosphere assemblies [18]. Two-photon excitation fluorescence of Cy5 dye by dipole and quadrupole modes of gold nanodisks has been studied using numerical simulations [15]. It is shown that fluorescence enhancement factors are higher upon coupling to both TPA and emission bands compared to those with coupled emission only. Recently, gold nanodisks have been used for experimental studies of simultaneous enhancement of excitation and emission of quantum dots [16]. The 10–20-fold higher luminescence brightness is reported in case of two-modal enhanced processes.

Achieving the highest possible fluorescence enhancement factors by simultaneous boosting of two-photon excitation and emission of a fluorophore depends on several factors: (i) strong enhancement of optical fields upon excitation of a low-energy LSPR mode in the near-infrared spectral region; (ii) existence of another LSPR mode in the visible range localized at the same hotspot; (iii) good spectral overlap of these modes with TPA and emission bands of a fluorophore; (iv) precise positioning of a fluorophore molecule at the hotspot. To design an efficiently coupled hybrid bionanosystem for fluorescence enhancement upon two-photon excitation, deep understanding of the involved processes and their relative contributions to the overall enhancement factor is essential.

Here, by using finite-difference time-domain (FDTD) simulations we study the fluorescence enhancement efficiency of eqFP670 near silver nanobars (NB), crystalline nanoparticles with a shape of square cuboid. Experimentally, these nanoparticles can be synthesized by silver ions reduction via polyol route [19,20,21]. The LSPR can be tuned by changing the particle size. Moreover, unlike nanorods, these faceted nanocrystals possess relatively sharp vertices, which induce strong field localization at the same hot spots for both dipole and quadrupole modes at longitudinal and transverse polarization of light. By changing length and aspect ratio of the particle, we analyze relative contributions from plasmon-induced two-photon excitation and emission enhancement and show that the fluorescence enhancement factor can be as high as $10^{3}$. Importantly, the single mode coupling regime with highly tuned near-field is found to significantly outperform the dual-mode coupling enhancement in this case. The plasmon-induced amplification of the fluorophore’s excitation rate becomes of utmost importance due to its quadratic dependence on light intensity, thus defining the overall fluorescence enhancement upon two-photon excitation.

## 2. Methods

To study fluorescence enhancement we used three-dimensional finite-difference time-domain method realized in the FDTD Solutions software package (Ansys). We employed a single emitter–single nanoparticle model. The nanobar is modeled by a block of silver oriented along the *x* axis, with edges and vertices rounded to 5 nm radius of curvature. The mesh step size of 2×2×2 nm is used in the region containing the particle and the emitter. Silver dielectric function is fitted to literature data [22]. The eqFP670 fluorophore molecule is represented by a point dipole with the emission wavelength $\lambda_{em}$ = 670 nm, corresponding to the maximum of the emission band of the protein. We also use the emitter’s intrinsic quantum yield value of $\varphi^{0}=0.06$. The system models an aqueous solution, so the surrounding medium is represented by the constant refractive index n=1.33. The light from a plane wave source propagates along the axis *z*, with linear polarization being either parallel or perpendicular to the axis *x*.

The fluorescence signal of an isolated fluorophore depends on the collection efficiency *k*, the excitation rate $\gamma_{exc}^{0}$ and the intrinsic quantum yield $\varphi^{0}$:

(1) $$\Gamma_{fl}^{0}=k\gamma_{exc}^{0}\varphi^{0}.$$

The fluorescence quantum yield is defined as a ratio between the radiative relaxation rate constant and the total relaxation rate constant, which accounts for nonradiative processes:

(2) $$\varphi^{0}=\frac{\gamma_{r}^{0}}{\gamma_{r}^{0}+\gamma_{nr}^{0}}.$$

The presence of a nanoparticle affects both the excitation rate constant and the quantum efficiency of the fluorophore. The fluorescence enhancement factor can be calculated as a ratio between the fluorescence signals of a coupled system and an isolated fluorophore. Since within a two-photon absorption regime the excitation rate is proportional to the fourth power of the electric field at the position of the fluorophore, $\gamma_{exc}^{0}\propto {\left|E\right|}^{4}$, the fluorescence enhancement factor EF becomes

(3) $$EF=\frac{\Gamma_{fl}}{\Gamma_{fl}^{0}}=\frac{\gamma_{exc}}{\gamma_{exc}^{0}}\frac{QE}{\varphi^{0}}=\frac{{\left|E\right|}^{4}}{|E_{0}{|}^{4}}\frac{QE}{\varphi^{0}}$$

The efficiency of a coupled emitter–antenna system can be found using the following relation [23]:

(4) $$QE=\frac{P_{r}/P_{r}^{0}}{P_{r}/P_{r}^{0}+P_{loss}/P_{r}^{0}+\left[1-\varphi^{0}\right]/\varphi^{0}},$$

where $P_{r}$ is the power radiated to the far field, $P_{r}^{0}$ is the power emitted by the fluorophore in the absence of the nanoparticle, and $P_{loss}$ accounts for the Ohmic losses inside the metal.

In this work, the fluorescence enhancement factor is calculated in two steps using Equation (3). First, a local distribution of electric field near Ag NB is calculated upon illumination at the wavelength of the longitudinal dipole resonance mode. Then, a classical point dipole, representing the fluorophore molecule, is placed at a particular location near the particle, and both the power, emitted by the dipole $P_{r}^{0}$, and the power, transmitted to the far-field $P_{r}$, are calculated and the corresponding value of EF is found.

## 3. Results and Discussion

The efficiency of multi-modal two-photon excitation fluorescence enhancement by Ag NB is studied using the fluorescent eqFP670 protein.

**Figure 1:**
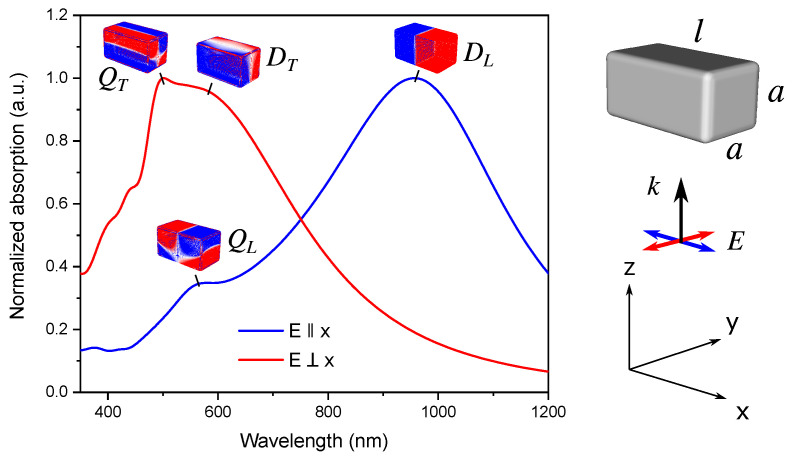
Normalized extinction of Ag nanobar with *l* = 180 nm and *a* = 100 nm upon illumination with light polarized parallel (blue) and perpendicular (red) to the long axis of the particle. The charge distribution plots show the corresponding LSPR modes at particular wavelengths: D and Q stand for the dipole and quadrupole modes; indices L and T denote the longitudinal and transverse polarization; also shown are the dimensions of the particle, the light propagation direction, and polarization.

The LSPR spectrum of Ag NB exhibits two distinct dipole resonances, which can be excited by light with different polarization with respect to the long axis of the NB. For large enough particles, quadrupole (and higher order) modes become visible at shorter wavelengths. The normalized extinction spectra of Ag NB together with the calculated charge distributions are presented in Figure 1. The quadrupole modes in the nanobar are characterized by opposite charges across the short axis of the particle, even at longitudinal polarization. This is in contrast to the case of thin nanorods, where quadrupole and higher order modes arise at longitudinal polarization and correspond to charge oscillations along the particle.

**Figure 2:**
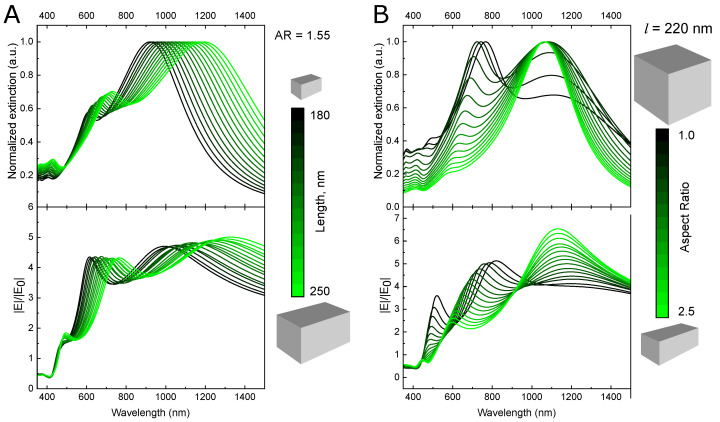
Spectral tunability of Ag nanobar’s LSPR modes. Normalized extinction spectra (top) and electric field enhancement (bottom) for the Ag nanobar of various lengths and the constant aspect ratio of 1.55 (**A**) and various aspect ratios at the constant particle length of 220 nm (**B**).

Spectral positions of LSPR modes can be tailored by tuning the size of the NB, as indicated in Figure 2. The position of the long-wavelength absorption peak, which corresponds to the longitudinal mode, is defined by the length *l* of the particle. Thus, by changing *l* from 180 to 250 nm, while keeping the aspect ratio equal to 1.55, the peak is shifted from 900 to 1200 nm.

By changing the nanobar’s width *a* at constant *l* (or, equally, changing the aspect ratio), the position of the quadrupole peak can be independently adjusted to match the emission band of the fluorophore (see Figure 2B).

The highest amplification of the electric field is observed near vertices of the nanobar (see Figure S1). According to Equation (3), the strong fluorescence enhancement requires a high electric field at the fluorophore’s TPA wavelength. Thus, it is important to know an exact spectral position of the maximum optical near fields. The calculated wavelength dependencies of the electric field |E|/|E${}_{0}$| at the point located 5 nm away from the corner of the NB are presented in Figure 2. As it can be seen, increasing the aspect ratio of the nanobar leads to the stronger electric field enhancement at the wavelength of the dipole LSPR mode, while the short-wavelength peak becomes decreased and blue-shifted.

**Figure 3:**
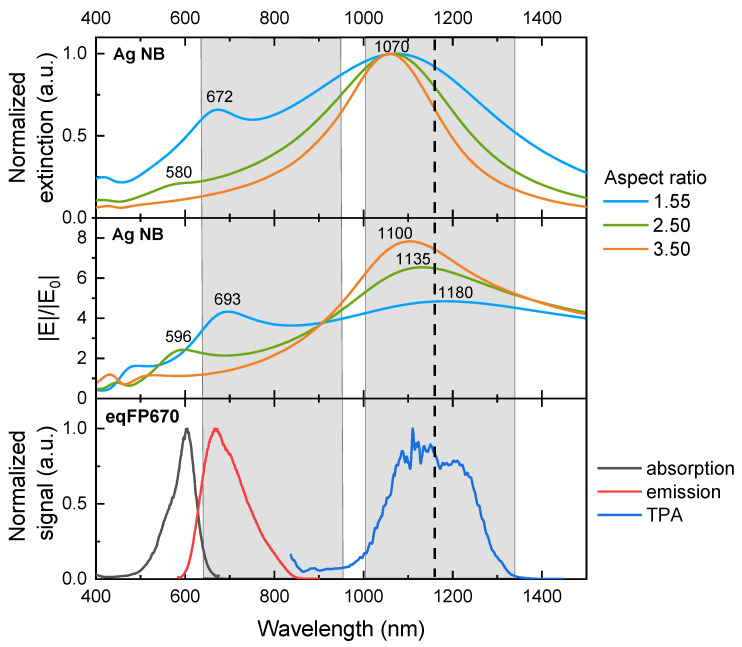
Spectral properties of the system under study. Top and middle: normalized extinction and electric field enhancement near Ag NB with l=220 nm and various aspect ratios. Bottom: normalized absorption (black), emission (red) and two-photon absorption (blue) spectra of eqFP670 [5]. The dashed line marks the irradiation wavelength. The grey regions demonstrate the two biological transparency windows.

To analyze the impact of excitation and emission enhancement on the overall fluorescence enhancement factor of eqFP670, we select particles with the length of 220 nm and various aspect ratios of 1.55, 2.50, and 3.50 for further studies. Figure 3 shows that all three particles have a good spectral overlap of the dipole-induced electric field near 1100 nm with the two-photon absorption band of eqFP670. At the same time, the local field enhancement is stronger for particles with a higher aspect ratio. Regarding the quadrupole resonance, only the particle with an aspect ratio of 1.55 has a good spectral overlap with the fluorophore’s emission. In other words, only the NB with an aspect ration of 1.55 supports dual-mode fluorescence enhancement. The question then arises, which system provides better fluorescence enhancement—the one with dual-mode coupling, or with higher field amplification?

**Figure 4:**
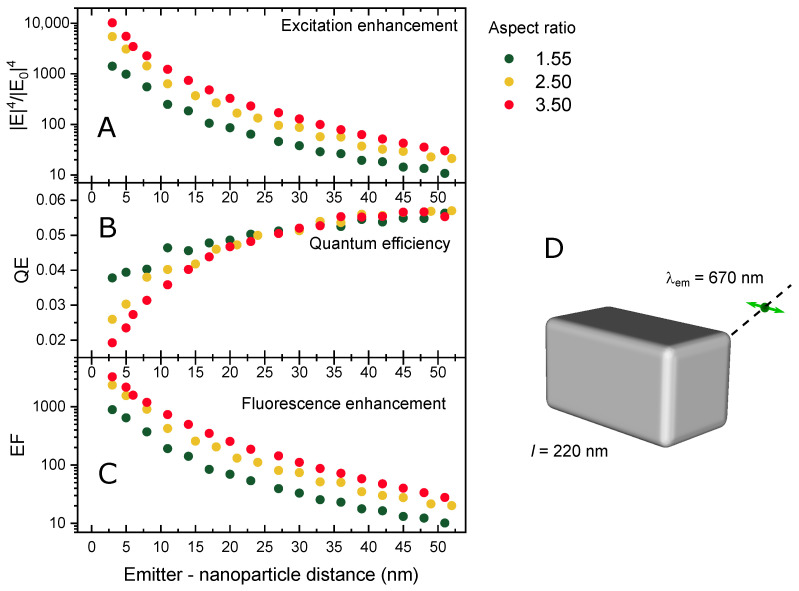
Two-photon excitation rate enhancement (**A**), quantum efficiency (**B**), and two-photon fluorescence intensity enhancement (**C**) as a function of the distance between the fluorophore and the NB along the diagonal for the nanobars with various aspect ratios; schematics of the emitter–nanobar configuration (**D**). The dashed line shows the scanning direction and the green arrow indicates the emitter’s polarization.

To address this question, we study how the excitation rate enhancement and the quantum efficiency change with the emitter–particle distance. We place the point dipole emitting light at $\lambda_{em}=670$ nm, which is polarized parallel to the long axis of the particle, at various positions along the particle’s diagonal and calculate QE and EF at every spot. The results are presented in Figure 4. As expected, the two-photon excitation rate, calculated as the fourth power of the electric field enhancement, quickly grows as the emitter approaches the Ag NB, reaching values of 10${}^{3}$–10${}^{4}$ at 3 nm distance. The particles with the higher aspect ratio demonstrate higher excitation rates.

The quantum efficiency of the hybrid system, on the other hand, exhibits the opposite behavior: QE tends to the value of an isolated emitter $\varphi^{0}=0.06$ at long separation distances, while it reduces at shorter distances due to nonradiative energy transfer to the metal. For NBs with stronger quadrupole mode, which overlaps with protein’s emission, this effect is mitigated by the enhanced stimulated emission (Purcell effect). Therefore, the QE for the NB with the aspect ratio of 1.55 is about two times higher than that of the NB with the aspect ratio of 3.5 at 3 nm separation.

In the one-photon absorption regime, the interplay between the excitation enhancement and the quenching effect results in a nonmonotonic dependence of the fluorescent enhancement factor on the distance with a maximum, which is usually observed at 5–10 nm separation between the molecule and the plasmonic nanoparticle [11,24]. In case of two-photon absorption, the effect of the excitation rate enhancement can still dominate at smaller separation distances due to its strong power dependence on local electric field. For the Ag NB—eqFP670 system, we observe no maximum on the calculated distance dependence of EF at least down to 3 nm separation. This result is consistent with the previous results [14,15,16]. The calculated fluorescence enhancement factor EF reaches a value of $10^{3}$, being higher for the particles with the higher aspect ratio. Increasing the separation quickly diminishes the enhancement—it drops by an order of magnitude at 15 nm distance.

The data obtained for eqFP670 placed at 3 nm distance to the corner of the nanobars with various aspect ratios are presented in Table 1.

Increasing the NB’s aspect ratio reduces the antenna quantum efficiency QE due to a blue shift of the quadrupole mode away from the eqFP760 emission band. At the same time, it leads to a stronger optical field at the TPA wavelength $\lambda_{exc}$ = 1180 nm. Since the fluorescence enhancement factor depends linearly on QE, but has fourth-power dependence on the amplification of the local electric field, the overall strong fluorescence enhancement is observed. It should be noted that at experimental conditions, all possible orientations of the NB with respect to the incident light polarization will be realized, thus the overall enhancement factor will be lower. Additionally, depending on a protein immobilization scheme the orientation of its dipole moment with respect to the particle can be different, which can also significantly affect the EF.

To summarize, for the hybrid system of the fluorescent eqFP670 protein placed close to the vertex of a silver NB with its dipole moment parallel to the long axis of the nanoparticle, the dual-mode coupling to both TPA and emission bands of the fluorophore is found to be less important than the local field amplification at the TPA wavelength. In this case, the efficiency of fluorescence enhancement is primarily defined by the fluorophore’s excitation rate, whereas coupling to the emission band has lower impact on the total enhancement factor. We expect that these findings are applicable to nanoparticles with other geometries provided that they exhibit reasonably strong localization of electric field at wavelengths coinciding with TPA bands of fluorophores. In the future studies, various shapes of nanoparticles are to be considered in order to gain simultaneous enhancement from both two-photon excitation and stimulated emission, while also keeping the near-field as high as possible.

## 4. Conclusions

By using FDTD numerical simulations, we show that silver nanobars can be used for efficient fluorescence enhancement of near-infrared fluorescent proteins upon two-photon excitation. The amplified optical near field is localized close to vertices of the nanobar particles upon excitation of both dipole and quadrupole LSPR modes. The resonance frequencies of these modes can independently be tuned by changing width and length of the particles.

We reveal that the higher NB’s aspect ratio leads to the stronger localized electric field upon excitation of the longitudinal dipole resonance mode. This allows us to reach enhancement factors of up to 10${}^{3}$ in the case of the fluorescent eqFP670 protein, despite the lack of the spectral overlap between the quadrupole LSPR mode and the emission peak of the fluorophore. We conclude that upon nonlinear absorption the efficiency of fluorescence enhancement is primarily defined by the fluorophore’s excitation rate due to its quadratic dependence on light intensity, whereas coupling to the emission band has lower impact on the total enhancement factor. The highly tuned design of the hybrid bionanosystem studied in this work can be used for developing bright near-infrared fluorescent probes for deep tissue bioimaging upon two-photon excitation. Our findings also have wider impact on the rational design of plasmonic nanostructures with reasonably strong localization of electric field at wavelengths coinciding with nonlinear absorption bands of fluorophores for two-photon excitation fluorescence enhancement.

## Figures and Tables

**Table 1:** Characteristics of the two-photon excitation fluorescence enhancement of eqFP670 placed at 3 nm to the corner of the Ag nanobar with various aspect ratios.

Aspect Ratio	|E|${}^{4}$/|E${}_{0}$|${}^{4}$	QE	QE/$\varphi^{0}$	EF
1.55	1418	0.038	0.63	893
2.50	5465	0.026	0.43	2361
3.50	10,190	0.019	0.32	3264

## Supplementary Materials

The following are available online at https://www.mdpi.com/article/10.3390/nano11123334/s1, Figure S1: Electric field distribution plots near the Ag nanobar upon excitation of different LSPR modes.

## Abbreviations

The following abbreviations are used in this manuscript:

EF	Enhancement Factor
FDTD	Finite Difference Time Domain
LSPR	Localized Surface Plasmon Resonance
NB	Nanobar
