# Supplementary material for: Efficiency of Plasmon-Induced Dual-Mode Fluorescence Enhancement upon Two-Photon Excitation
Source: Nanomaterials (Basel). 2021 Dec 8;11(12):3334. doi: 10.3390/nano11123334 (PMC8705879; doi:10.3390/nano11123334)
Supplement: Supplementary file 1 [file nanomaterials-11-03334-s001.zip › nanomaterials-1461947-supplementary.pdf]

Supplementary Materials: Efficiency of plasmon-induced dual-mode fluorescence enhancement upon two-photon excitation

Maria A. Shokova and Vladimir E. Bochenkov*

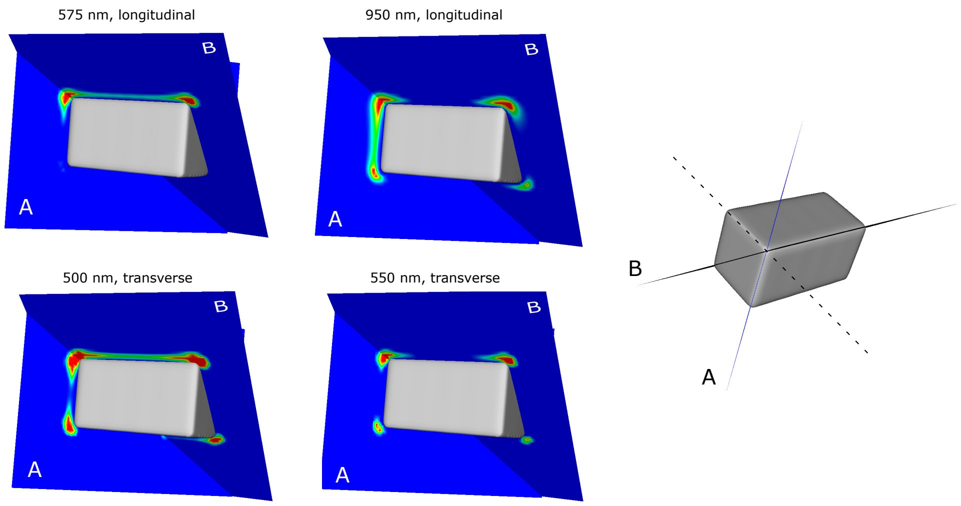

Figure S1. Electric field distribution plots near the Ag nanobar upon excitation of different LSPR modes. Top row: longitudinal polarization, bottom row: transverse polarization. Left column: quadrupolar mode, right column: dipolar mode. The electric field is shown by two monitor planes, which bisect the particle, as indicated in the diagram on the right. The enhanced field is localized near the vertices in all cases.
